# Supplementary material for: Identification and functional analysis of non-coding regulatory small RNA FenSr3 in Bacillus amyloliquefaciens LPB-18
Source: PeerJ. 2023 May 15;11:e15236. doi: 10.7717/peerj.15236 (PMC10194069; doi:10.7717/peerj.15236)
Supplement: Supplemental Information 4 [file peerj-11-15236-s004.zip › KO/CK-vs-T1_map/map04360.html]

KEGG PATHWAY: Axon guidance - Reference pathway


|  |  |
| --- | --- |
| **Axon guidance - Reference pathway** |  |

[
Pathway menu
| Organism menu
| Pathway entry
| Show description
| User data mapping
]

Axon guidance represents a key stage in the formation of neuronal network. Axons are guided by a variety of guidance factors, such as netrins, ephrins, Slits, and semaphorins. These guidance cues are read by growth cone receptors, and signal transduction pathways downstream of these receptors converge onto the Rho GTPases to elicit changes in cytoskeletal organization that determine which way the growth cone will turn.

|  |  |  |
| --- | --- | --- |
| Reference pathway | 184% 150% 122% 100% 82% 67% 55% | 图片下载 |
